# Factors influencing the use of different methods of consent in a randomized acute stroke trial: The Third International Stroke Trial (IST-3)

**DOI:** 10.1177/17474930211037123

**Published:** 2021-08-10

**Authors:** Richard I Lindley, Ingrid Kane, Geoff Cohen, Peter AG Sandercock

**Affiliations:** 1 Westmead Applied Research Centre, University of Sydney, Sydney, Australia; 2 Neurological & Mental Health Division, The George Institute for Global Health, Sydney, Australia; 3 Department of Stroke Medicine, Royal Sussex Country Hospital, Sussex University Hospitals Trust, Brighton, UK; 4 Centre for Clinical Brain Sciences, The University of Edinburgh, Edinburgh, UK

**Keywords:** Informed consent, acute stroke therapy, aphasia, thrombolysis, clinical trial, ischemic stroke

## Abstract

**Background:**

Obtaining informed consent in people with acute stroke is complex since many, as a direct result of their stroke, lose capacity to make important decisions. Furthermore, reperfusion interventions are time dependent necessitating rapid consent. We developed four different consent approaches to facilitate recruitment of a broad range of patients in the Third International Stroke Trial (IST-3).

**Aims:**

To describe the clinical characteristics of patients recruited by different consent methods and the association between these methods and time from stroke onset to randomization.

**Methods:**

IST-3 was a randomized controlled trial of thrombolysis for acute ischemic stroke. Clinicians could use one of four consent procedures: written consent, witnessed consent, assent, or a waiver of consent. We analyzed the relationship between consent procedure and baseline variables. The effect of consent procedure on delay time from onset to randomization was determined using analysis of variance to adjust for confounding effects.

**Results:**

Of the 3035 patients recruited, the method of consent was known for 3034 (99.9%), and it was written in 985 subjects (32.5%), witnessed verbal consent in 280 (9.2%), assent by relative in 1727 (56.9%), and waiver of consent in 42 subjects (1.4%). Assent was required in 63.4% for those presenting 0–3 h from stroke onset (written consent in 25.3%). Patients with more severe neurological deficits (or with a non-lacunar hemispheric stroke syndrome) were less likely to give written consent. Mean delay between onset and randomization varied significantly between consent types (one-way analysis of variance: F = 15.7 on 3 df, p < 0.0001) (longest at 4.06 h for signed consent and 3.46 h for waiver of consent).

**Conclusions:**

Acute stroke trials requiring written informed consent would result in substantial selection bias. Flexible consent methods will ensure a broad range of patients are recruited, enabling trial results to be widely generalizable.

**Registration:** This study’s registered number is ISRCTN25765518.

## Introduction

Obtaining informed consent in people with acute stroke is complex since many, as a direct result of their stroke, lose capacity to make important decisions.^1^ Furthermore, some interventions, such as thrombolytic therapy or thrombectomy, are time dependent, with a rapid decline in treatment efficacy with increasing delay from stroke onset.^2,3^ If we are to evaluate new stroke treatments, such as new regimes for thrombolysis (by dose or agent) or thrombectomy (by device or adjuvant methods), these will also likely have important loss of treatment efficacy with increasing delay from stroke onset. Although many participants in acute stroke randomized controlled trials have been recruited with consent obtained from a relative (or “person responsible”) as permitted in each jurisdiction,^4^ exclusion of those, for example, with aphasia, has been common for post-acute interventions.^5^ We previously published the consent procedures from the first 300 participants^1^ and now report how consent was recorded from the full Third International Stroke Trial (IST-3) data set to quantify the use of approved consent methods in this large acute treatment trial.

## Aims

To describe the clinical characteristics of patients recruited by different consent methods and the association between these methods and time from stroke onset to randomization.

## Methods

Participants were eligible for IST-3 if they did not have definite contraindications for thrombolysis, and the clinicians considered thrombolysis promising but unproven. Trial treatment had to be commenced within 6 h of clinically definite stroke onset, and after brain imaging had reliably excluded intracranial hemorrhage or structural brain lesions that could mimic stroke.^6,7^ The patient information leaflet was developed with consumer involvement^8^ and most ethics committees for the 156 centers around the world allowed for consent to be obtained from a relative or “person responsible,” with some also permitting a waiver of consent mechanism.

After appropriate consent, and prior to randomization, key baseline data were collected (via a telephone voice-activated or a secure web-based randomization system), including the key neurological deficits and demographic data. At seven days (or earlier if the patient had been discharged or had died), data were collected on how consent was recorded. On this form, under a heading titled “How was consent obtained?” the response options were “Patient signed form; patient gave verbal consent; assent by relatives; waiver of consent (if waiver give reason).” Other items on this seven-day form included: medical treatment in hospital, the final diagnosis of the stroke event that led to randomization, side effects of treatment, brain imaging, complications, and discharge (or seven-day) functional status. Six-month vital status and functional outcome was obtained at six months by postal questionnaire or telephone interview. In this paper, “Assent by relatives” covers all the approved ways in which a participant could be entered into the trial, as approved by a relative, a “person responsible” or other legally established surrogate.

We tabulated type of consent by age, stroke severity (National Institutes of Health Stroke Scale (NIHSS)), conscious level (Glasgow Coma Scale), neurological impairment, stroke subtype (defined by the Oxfordshire Community Stroke Project (OCSP) classification),^9^ early (seven-day) mortality, six-month functional outcome, and delay from stroke onset to randomization. Chi-squared tests were used to examine whether the different types of consent were associated with any of these variables. We also used analysis of variance (ANOVA) to investigate whether the time delay from onset to randomization was associated with consent type after adjusting for the strong associations of age and stroke severity with consent type. The protocol was approved by the Multicentre Research Ethics Committees (in Scotland, reference MREC/99/0/78) and by local ethical committees. The registered number of this study is ISRCTN25765518.

## Role of the funding source

The sponsors and funding agencies for the study (see Sandercock et al.^6^ for full list of funders) had no role in data collection, data storage, data analysis, preparation of this report, or the decision to publish. The corresponding author had full access to all the data in the study and had final responsibility for the decision to submit for publication.

## Results

Data on method of consent were available for 3034 out of 3035 patients. The missing value was in a patient with no seven-day form, but who was known to be alive at six-month follow-up, so was thereby alive at seven days. The commonest record of the consent procedure was assent (56.9%), followed by signed consent by patient (32.5%), verbal consent by patient (9.2%), with few patients randomized with a waiver of consent procedure (1.4%) (Table 1).

**Table 1.** Baseline characteristics by consent approach in the Third International Stroke Trial

	Consent type								
	Written		Verbal		Assent		Waiver		Total
Characteristic	n	(%)	n	(%)	n	(%)	n	(%)	n
Age									
<80	590	(41.6)	137	(9.7)	674	(47.6%)	16	(1.1%)	1417
>80	395	(24.4)	143	(8.8)	1053	(65.1)	26	(1.6)	1617
Χ^2 ^= 112, p < 0.0001									
NIHSS									
0–5	434	(70.9)	52	(8.5)	123	(20.1)	3	(0.5)	612
6–18	530	(29.6)	189	(10.6)	1046	(58.5)	24	(1.3)	1789
19+	21	(3.3)	39	(6.2)	558	(88.2)	15	(2.4)	633
Χ^2 ^= 722, p < 0.0001									
Glasgow Coma Scale									
<10	3	(2.0)	8	(5.3)	132	(87.4)	8	(5.3)	151
10–11	13	(3.0)	9	(2.1)	402	(93.5)	6	(1.4)	430
12–13	57	(9.7)	33	(5.6)	485	(82.6)	12	(2.0)	587
14–15	912	(48.9)	230	(12.3)	708	(37.9)	16	(0.9)	1866
Χ^2 ^= 795, p < 0.0001									
Dysphasia									
Yes	266	(16.8)	122	(7.7)	1167	(73.7)	29	(1.8)	1584
No	715	(51.6)	155	(11.2)	506	(36.5)	9	(0.6)	1385
Cannot assess	4	(6.2)	3	(4.6)	54	(83.1)	4	(6.2)	65
Χ^2 ^= 470, p < 0.0001 (ignoring “Cannot assess”)									
Visuospatial disorder									
Yes	260	(24.0)	106	(9.8)	708	(65.4)	8	(0.7)	1082
No	666	(49.7)	128	(9.5)	536	(40.0)	11	(0.8)	1341
Cannot assess	59	(9.7)	46	(7.5)	483	(79.1)	23	(3.8)	611
Χ^2 ^= 179, p < 0.0001 (ignoring “Cannot assess”)									
Motor deficit									
Yes	710	(27.5)	247	(9.6)	1583	(61.3)	41	(1.6)	2581
No	275	(60.7)	33	(7.3)	144	(31.8)	1	(0.2)	453
Χ^2 ^= 197, p < 0.0001									
Stroke subtype									
TACI	149	(11.4)	115	(8.8)	1017	(77.9)	25	(1.9)	1306
PACI	452	(39.4)	111	(9.7)	572	(49.9)	11	(1.0)	1146
LACI	229	(69.0)	35	(10.5)	65	(19.6)	3	(0.9)	332
POCI	150	(61.2)	19	(7.8)	73	(29.8)	3	(1.2)	245
Other	5	(100.0)							5
Χ^2 ^= 625, p < 0.0001 (excluding Other)									

NIHSS: National Institutes of Health Stroke Scale; TACI: total anterior circulation infarct; PACI: partial anterior circulation infarct; LACI: lacunar infarct; POCI: posterior circulation infarct.

The distribution of consent type differed significantly between those aged 80 years and younger compared to the over 80 year olds, with more assent and less written consent in the over 80 year olds (p < 0.0001). Stroke severity, whether measured by the NIHSS, Glasgow Coma Scale, or by stroke subtype (OCSP) also influenced consent type (p < 0.0001 for all three tests), with assent increasingly common for those with increased stroke severity (Table 1). Consent signed by the patient was least likely with total anterior circulation infarct (11.4%) and most likely after lacunar stroke (69%).

Patients who provided written signed consent had the best predicted outcome, as calculated by the Konig score (58% chance of good outcome), as well as the best actual outcome (61% alive and independent).^10^ Of the patients in whom consent was obtained by assent, 25% had a predicted good outcome, and at six months, 22% were alive and independent. Actual outcomes by consent approach are shown in Table 2.

**Table 2.** Important outcomes by consent approach in the Third International Stroke Trial

Consent type									
	Written		Verbal		Assent		Waiver		Total
Died within seven days									
Yes	955	(34.6%)	263	(9.5%)	1511	(54.7%)	35	(1.3%)	2764
No	30	(11.1%)	17	(6.3%)	216	(80.0%)	7	(2.6%)	270
Χ^2 ^= 75.2, p < 0.0001									
Status at six months									
Alive and independent	601	(55.3%)	94	(8.6%)	383	(35.2%)	9	(0.8%)	1087
Dead or dependent	384	(19.7%)	186	(9.6%)	1344	(69.0%)	33	(1.7%)	1947
Χ^2 ^= 416.2, p < 0.0001									
Delay from onset to randomization (h)									
0–3	214	(25.2%)	83	(9.8%)	538	(63.4%)	14	(1.6%)	
3–4.5	377	(32.1%)	103	(8.8%)	676	(57.5%)	20	(1.7%)	
4.5–6	394	(39.1%)	93	(9.2%)	512	(50.8%)	8	(0.8%)	
Mean	4.06		3.83		3.75		3.46		
SD	1.19		1.24		1.2		1.25		
SE	0.04		0.07		0.03		0.19		
One-way ANOVA F = 15.7 on 3 df, p < 0.0001)									
Total N	985	(32.5%)	280	(9.2%)	1727	(56.9%)	42	(1.4%)	3034

ANOVA: analysis of variance; SD: standard deviation; SE: standard error.

Mean delay between onset and randomization varied significantly between consent types (one-way ANOVA: F = 15.7 on 3 df, p < 0.0001) (longest at 4.06 h for signed consent and 3.46 h for waiver of consent), but this difference was not statistically significant after allowing for age and NIHSS stroke severity (one-way ANOVA: F = 1.2 on 3 df, p = 0.3068).

## Discussion

IST-3 included a large proportion of older people, many of whom had a severe stroke, and we have demonstrated that only one third were able to sign to confirm their consent to the trial. A majority of participants entered the trial with an assent confirmation. This illustrates the importance of designing mechanisms of consent to allow appropriate inclusion into randomized controlled trials. Given that a majority of participants were unable to contribute to the consent procedure in the acute phase of stroke, it is important that researchers consider appropriate consumer co-design to ensure people agree with the research ideas and provide guidance on appropriate consent materials.^8^ Co-design will also help ensure the most ethical approach and reduce unacceptable risks for this vulnerable patient group. Our data also reinforce the importance of flexible consent. If patients, suddenly mentally incompetent due to illness, are excluded from research, it reduces the likelihood of future advances of care for many acute disorders as well as stroke. This is particularly important for those with very severe stroke who are often aphasic or have reduced conscious level. There is also evidence to suggest that even in patients who are assessed to have capacity to consent to treatment, they may not recall this information afterwards.^11^ Capacity for consent takes time, and in the acute situation, this can be challenging and distressing for patients potentially delaying time critical treatments.^11^ We would recommend that researchers planning further acute stroke intervention studies invest time and planning for the “person responsible” information leaflets, as assent could be the commonest type of consent procedure.

The IST-3 data illustrate the potential dangers of selection bias if studies are only approved for those providing signed consent. In acute ischemic stroke, such patients have a far more favorable outcome, but may only represent about a third of all ischemic stroke. Tu et al. have previously published a real case example of such selection bias in the context of low-risk research (a stroke clinical register).^12^

Although some of our centers had approval for “waiver of consent,” this is not always allowed in different legal systems. During the trial, it also became obvious that waiver of consent had limited utility, as a witness was usually required to provide accurate details of the time of stroke onset, and other key eligibility criteria. Although waiver of consent was used rarely in IST-3 (about 1%), we are uncertain of the value of this, as many of our centers were probably not permitted to use this procedure. Overall, this contributed only a few participants. A survey of participants recruited in the ESCAPE trial found that those recruited with a deferred consent process generally disagreed with the process so this remains a contentious issue.^13^

Strengths of our study include the prospective collection of consent method in a large-scale trial, completed in 156 centers from 12 countries. Our prior consumer involvement had led to patient-friendly trial materials.^8^ Weaknesses include our streamline trial processes did not include additional baseline data that would have been interesting, such as door to needle time, or how many Human Research Ethics Committees determined that waiver of consent was not permitted in that jurisdiction.

Given the prevalence of lack of mental capacity in acute stroke, we commend flexible approaches as discussed by Silbergleit and Dickert,^14^ and guidelines in Australia, that recommend flexibility in consent procedures and that consent “should not be unnecessarily long or detailed, even for complex interventional research.”^15^

## References

[1] Kane I Lindley R Lewis S and Sandercock P on behalf of the IST-3 Collaborative Group . Impact of stroke syndrome and stroke severity on the process of consent in the Third International Stroke Trial. Cerebrovas Dis 2006; 21: 348–352. 10.1159/000091541 16490945

[2] Emberson J Lees KR Lyden P , et al. Effect of treatment delay, age, and stroke severity on the effects of intravenous thrombolysis with alteplase for acute ischaemic stroke: a meta-analysis of individual patient data from randomised trials. Lancet 2014; 384: 1929–1935. 25106063 10.1016/S0140-6736(14)60584-5 PMC4441266

[3] Saver JL Goyal M van der Lugt A , et al. Time to treatment with endovascular thrombectomy and outcomes from ischemic stroke: a meta-analysis. JAMA 2016; 316: 1279–1289. 27673305 10.1001/jama.2016.13647

[4] Ali M Bath PM Lyden PD Bernhardt J Brady M . Representation of people with aphasia in randomized controlled trials of acute stroke interventions. Int J Stroke 2014; 9: 174–182. 23506193 10.1111/ijs.12043

[5] Wray F Clarke D Forster A . Post-stroke self-management interventions: a systematic review of effectiveness and investigation of the inclusion of stroke survivors with aphasia. Disabil Rehabil 2018; 40: 1237–1251. 28271913 10.1080/09638288.2017.1294206

[6] Sandercock PAG Wardlaw JM Lindley RI , et al. The benefits and harms of intravenous thrombolysis with recombinant tissue plasminogen activator within 6 h of acute ischaemic stroke (the third international stroke trial [IST-3]): a randomised controlled trial. Lancet 2012; 379: 2352–2363. 22632908 10.1016/S0140-6736(12)60768-5 PMC3386495

[7] Sandercock P Lindley R Wardlaw J , et al. Statistical analysis plan for the third International Stroke Trial (IST-3); part of a ‘threat’ of reports of the trial. Int J Stroke 2012; 7: 186–187. 22405276 10.1111/j.1747-4949.2012.00782.x

[8] Koops L Lindley RI . Thrombolysis for acute ischaemic stroke: consumer involvement in design of new randomised controlled trial. Br Med J 2002; 325: 415–417. 12193356 10.1136/bmj.325.7361.415 PMC119434

[9] Bamford J Sandercock P, Dennis M, Burn J Warlow C , et al. Classification and natural history of clinically identifiable subtypes of cerebral infarction. Lancet 1991; 337: 1521–1526. 1675378 10.1016/0140-6736(91)93206-o

[10] Konig IR Ziegler A Bluhmki E , et al. Predicting long-term outcome after acute ischemic stroke: a simple index works in patients from controlled clinical trials. Stroke 2008; 39: 1821–1826. 18403738 10.1161/STROKEAHA.107.505867

[11] Akinsanya J Diggory P Heitz E Jones V . Assessing capacity and obtaining consent for thrombolysis for acute stroke. Clin Med 2009; 9: 239–241. 10.7861/clinmedicine.9-3-239 PMC4953610 19634386

[12] Tu JV Willison DJ Silver FL , et al. Impracticability of Informed Consent in the Registry of the Canadian Stroke Network. N Engl J Med 2004; 350: 1414–1421. 15070791 10.1056/NEJMsa031697

[13] Shamy MCF Dewar B Chevrier S , et al. Deferral of Consent in Acute Stroke Trials. Lessons from the ESCAPE Trial. Stroke 2019; 50: 1017–1020. 30869570 10.1161/STROKEAHA.118.024096

[14] Silbergleit R Dickert NW . Context and principles must drive alternatives to consent in emergency research. Lancet Neurol 2020; 19: 968–969. 33098756 10.1016/S1474-4422(20)30367-7

[15] Commonwealth of Australia. *National statement on ethical conduct in human research 2007* (updated 2018). Canberra: The National Health and Medical Research Council, the Australian Research Council and Universities Australia, 2018.

